# Evaluation of the Home Environment Assessment for the Visually Impaired (HEAVI): an instrument designed to quantify fall-related hazards in the visually impaired

**DOI:** 10.1186/s12877-016-0391-2

**Published:** 2016-12-09

**Authors:** Bonnielin K. Swenor, Andrea V. Yonge, Victoria Goldhammer, Rhonda Miller, Laura N. Gitlin, Pradeep Ramulu

**Affiliations:** 1 Dana Center for Preventive Ophthalmology, Wilmer Eye Institute, Johns Hopkins University, 600 N Wolfe St, Wilmer Room 116, Baltimore, MD 21287 USA; 2 Center for Innovative Care in Aging, School of Nursing, Johns Hopkins University, Baltimore, MD USA

**Keywords:** Falls, Home assessment, Visual impairment

## Abstract

**Background:**

To (1) develop and refine the Home Environment Assessment for the Visually Impaired (HEAVI), and (2) determine the interrater reliability of this instrument, which was designed to quantify the number of fall-related hazards in the homes of individuals with visual impairment.

**Methods:**

Twenty homes of community-dwelling adults were included in this study. Each home was graded by an occupational therapist (OT) and two non-expert (NE) graders. Seventy-three HEAVI items were evaluated in eight rooms, for a total of 185 potential hazards per home (some items were assessed in multiple rooms). Pairwise and three-way agreement between graders was evaluated at the item, room, and home level using Krippendorff’s alpha and intraclass correlation coefficients (ICC). Additionally, the most hazardous home locations and items were determined by comparing the mean and standard deviation of the number of hazards by room and grader.

**Results:**

Of the 73 items, 45 (62%) demonstrated at least moderate agreement overall and for each OT/NE pair (Krippendorff’s alpha >0.4), and remained in the final instrument (a total of 119 potential hazards per home as some items were assessed in multiple rooms). Of these 119 potential hazards, an average of 35.7, 33.2, and 33.3 hazards per home were identified by the OT and NE graders, respectively. Moderate to almost perfect agreement on the number of hazards per home and number of hazards per room, except the dining room, was found (ICCs of 0.58 to 0.93). Bathroom items were most often classified as hazards (>40% of items for all graders). The item classes most commonly graded as hazardous were handrails and lighting (>30% of items).

**Conclusion:**

Our results indicate that NE graders can accurately administer the HEAVI tool to identify fall-related hazards. Items in the bathroom and those related to handrails and lighting were most often identified as hazards, making these areas and items important targets for interventions when addressing falls.

## Background

Falls affect one third of adults over the age of 65 annually [1] and are the leading cause of fatal and nonfatal injuries in this age group [2]. Many older adults who fall develop fear of falling, and up to 40% will restrict their activities of daily living, leading to a decline in physical activity and social interactions [3]. Fall risk factors can include features of the home environment such as loose rugs, floor clutter, and poor lighting [4, 5], which are possible targets for fall interventions. The majority of falls occur in or near the home [6–8], highlighting the importance of developing and evaluating tools that assess fall risk in the home environment. As studies have shown that home modifications are effective in reducing falls, a reliable tool is important to inform such interventions [9–13].

The home environment may pose particular risk for older adults with visual impairments given difficulty with hazard perception. Impaired vision can manifest as visual field loss, decreased visual acuity, and/or decreased contrast sensitivity, and each is associated with increased fall risk [14–24]. In the United States, 2.4 million adults have low vision, and an additional 937,000 are blind; these numbers are projected to increase to 3.9 and 1.6 million, respectively, by the year 2020 [18]. The high prevalence of visual impairment, along with the important role that environmental hazards may play with regard to falls, point to a need for home assessment tools that detect hazards that are specifically problematic for older adults with vision loss.

Multiple home assessments have been developed to evaluate the home safety and fall risk of older individuals, though none have focused on individuals with visual impairments [25–29]. Many of these home assessments evaluate general indoor and outdoor lighting but do not adequately assess home hazards that are likely to be problematic for individuals with poor vision, such as lighting (wattage, placement, switch access, window coverings), color contrast, visual distractions, and glare [30]. There has also been an overreliance on subjective assessments of home environmental conditions in previous tools [30], as well as required grading by experts such as occupational therapists. Low vision rehabilitation has increasingly focused on home-based services, including evaluation and modification of the home environment; however, the lack of a standardized assessment for this population makes it impossible to compare outcomes of evaluations and interventions across practices. This represents an unmet need for a vision-centric home assessment tool that can be easily and reliably administered by individuals with varied training and areas of expertise [31].

This study reports on a novel tool, the Home Environment Assessment for the Visually Impaired (HEAVI), designed to identify specific hazardous items that may contribute to fall risk in persons with poor vision. The aim of this work was to: (1) develop and refine the HEAVI, and (2) determine the interrater reliability of this instrument. Items for the tool and the approach to measurement were derived from the literature and based on the Home Environmental Assessment Protocol (HEAP), an assessment for persons with dementia living at home that has been shown previously to have adequate content validity and interrater reliability [25]. Here, we assess the interrater reliability of HEAVI items based on agreement between an expert grader (a licensed occupational therapist with a clinical practice focused on assessing homes for safety) and two non-expert graders (non-occupational therapists without specialized training in environmental assessments). The goal of creating this tool is to support further research to identify which home hazards contribute most to falls, predict who is at greatest risk of falling, and develop targeted interventions for this population.

## Methods

### Study participants

The study protocol was approved by the Johns Hopkins Medicine Institutional Review Board. A convenience sample of 20 participants was recruited from the Baltimore/DC area from various sources including home- and clinic-based patients and word-of-mouth referrals, and informed consent was obtained from each participant. These participants were not recruited based on visual impairment status or overall health status.

### Development of the HEAVI

This home assessment tool adapted many items from the HEAP [25], but excluded items specific to individuals with dementia and caregivers (e.g., lack of safety latches on cabinet doors, lack of safety adaptations to oven/stove). A total of 18 items related to poor vision were initially added based on a literature review and the professional experience of an occupational therapist (OT) with expertise working with individuals with vision loss (VG). The literature review used search terms related to lighting, object contrast, stairs, and visual impairment; was conducted in PubMed and Google Scholar; and included literature from the past 30 years that was published in English. The references from sentinel work and conference proceedings in environmental modification, falls, and occupational therapy related to lighting, contrast, stairs, and visual impairment were reviewed for citations not captured by the original review. Additionally, relevant government documents were reviewed to identify safety guidelines for hazard classification cut points, such as stair riser height.

Items added to the HEAVI included 12 items assessing lighting and six assessing contrast between objects, which are particularly important for those with vision loss, as lack of appropriate lighting and contrast may make those with visual impairment at greater risk of falling .[32, 33]. Items were chosen and worded such that no specific training would be needed to complete the home assessment. Items were reviewed and refined twice based on review and discussion within the research team.

The 73 distinct items in the initial instrument were subcategorized into five classes: 1) handrails, 2) lighting, 3) floors, 4) furniture, and 5) other. These items were graded in up to eight rooms of the home: 1) entryway, 2) living room, 3) dining room, 4) kitchen, 5) bedroom, 6) bathroom, 7) stairs, and 8) hallways. Of the 73 distinct items, several (such as ambient lighting) were graded in multiple rooms, for a total of 185 possible hazards per home. Table 2 summarizes the items, shows the item subcategorization, defines the threshold for classifying items as hazardous, and outlines the potential rooms where each item was assessed.

### Training of graders

Prior to grading, the two non-expert graders received instruction on how to complete the home assessment from the OT expert grader (VG). The non-expert graders were both ophthalmic research technicians familiar with interacting with visually impaired patients, but with no prior experience in home assessment. This group was chosen, as we wanted to be able to assess a non-expert group of graders. The expert grader reviewed the home assessment tool with both non-expert graders, explained how each item should be assessed, and provided basic definitions of items on the form (e.g. what constitutes a threshold), as well as training on measuring items such as ambient lighting. This training lasted approximately 2 h. The OT confirmed with each non-expert grader that the assessment process was understood. All three graders independently assessed each room of the first test house. After the first home was assessed, all three graders discussed and compared their results. The OT discussed and explained the proper procedure(s) for each discrepancy grading between the two non-expert graders and the OT. A second house was assessed, and the same process was repeated, with each session lasting approximately 2 h. Neither of the homes used for training were included in these analyses. During the data collection of the 20 homes included in these analyses, the graders did not discuss or compare results.

### Home assessment procedures

A single home visit was scheduled for each study participant. Participants were instructed to arrange all rooms, including lighting, as they normally would, and asked not to clean or tidy prior to evaluation of the home. Study participants were typically in the home at the time of the visit, but not necessarily in the individual rooms being evaluated. For each home assessment, all three graders independently assessed the home and agreed on the specific entrances and rooms to evaluate, but each grader evaluated rooms independently and in separate rooms. There was no discussion to address whether each grader evaluated the same items within each room.

For each categorical item, answer choices included “Yes,” “No,” or “Not Assessed,” and items graded as “Yes” were classified as hazards. Clutter was defined as items on the floor, excluding furniture, that cover potential walkable space and was categorized as “None,” “1–25%,” “26–50%,” “51–75%,” or “76–100%.” Light intensity indoors and outdoors was measured in lux using a digital light meter (Dr. Meter model LX1330B). All size and distance measurements were rounded to the nearest half-inch. These continuous measures were then reclassified as hazardous or non-hazardous based on guidelines from the Americans with Disabilities Act [34], International Residential Code [35], Lighting Research Center at Rensselaer Polytechnic Institute [36], and Illuminating Engineering Society of North America [37]. Demographic information such as age (in years), race (white or African-American), years of education completed, cohabitation status (living alone or with others), marital status (married or single/divorced/widowed), and employment status (employed or retired/disabled/unemployed) were collected from all participants. Participants also provided information on their homes, including dwelling type (apartment or house), ownership status (rent or own), number of floors, and total number of rooms in the home. Home square footage was obtained from Zillow, an online real estate database (http://www.zillow.com), to characterize the size of the homes being graded.

### Statistical analyses

#### Refinement of the HEAVI

Item grading was aggregated to calculate: 1) the total number of rooms per home with each type of hazard (for example, the number of rooms with an ambient lighting hazard), 2) the total number of hazards in each room, and 3) the total number of hazards in the entire home. Interrater agreement was calculated for each of these variables by comparing responses from each of the non-expert graders with the expert grader, responses between the non-expert graders, and responses across all three graders as a three-way comparison. Agreement on the grading of each item was calculated using Krippendorff’s alpha, a standard measure of agreement that accommodates missing data [38]. Based on these results, the HEAVI was refined by removing items with poor agreement using criteria from Landis and Koch [39]: 1) poor agreement (<0.00), 2) slight agreement (0.00 – 0.20), 3) fair agreement (0.21 – 0.40), 4) moderate agreement (0.41 – 0.60), 5) substantial agreement (0.61 – 0.80), and 6) almost perfect agreement (0.81 – 1.00). Items with less than moderate agreement (≤0.40) were removed from the instrument prior to further analyses.

### Assessment of interrater agreement

Using the refined tool described above, we aimed to determine if individuals without specialized occupational therapy training can reliably use this instrument. To do so, intraclass correlation coefficients (ICC(2,1)) and 95% confidence intervals (CI) were used to calculate the absolute interrater agreement for each of the 20 homes. To do so, we aggregated the number of rooms with each hazard and number of hazards per room and home. Intraclass correlation coefficients were qualitatively evaluated using criteria from Landis and Koch [39], described above.

Bland-Altman plots were constructed to determine if agreement was constant across the number of noted hazards. [40] Lastly, the most hazardous home locations and item classes were determined by comparing the mean and standard deviation (SD) of the number of hazards by room and grader. All data were analyzed using Stata 14.0 (StataCorp, LP, College Station, TX).

## Results

This study includes 20 participants and their homes. The average age of study participants was 63.1 years (SD = 18.8). Approximately 25% were male, and 90% were white (Table 1). Most participants lived in a home (65%) as opposed to an apartment (35%), with a mean of 2.1 floors (SD = 0.9), 4.4 rooms (SD = 1.2), and 2,202 sq. ft. across residences.

**Table 1:** Demographics of participants and their homes. (*n* = 20 homes)

Variable	Result
Age, mean (SD)	63.1 (18.8)
Age, years, range	34–92
Male, n (%)	5 (25.0%)
Race, n (%)	
White	18 (90.0%)
African-American	2 (10.0%)
Education in years, mean (SD)	15.6 (2.6)
Lives alone, n (%)	8 (40.0%)
Married, n (%)	9 (45.0%)
Employed, n (%)	9 (45.0%)
Dwelling type, n (%)	
House	13 (65.0%)
Apartment	7 (35.0%)
Housing status, n (%)	
Own	16 (88.9%)
Rent	2 (11.1%)
Number of floors, n (SD)	2.1 (0.9)
Number of rooms, n (SD)	4.4 (1.2)
Housing size, square feet, mean (SD)	2,202 (877)^a^

^a^Data unavailable for seven homes

### Refinement of the HEAVI

Krippendorff’s alpha values were calculated for each item across all rooms to determine agreement between all three graders (Table 2). Levels of three-way agreement ranged from low levels of agreement (−0.06) to perfect agreement (1.00). Among the 73 items graded, 48 (66%) demonstrated moderate to perfect agreement (three-way Krippendorff’s alpha >0.4) [39], while 25 (34%) demonstrated fair to poor agreement and were not included in the final instrument. Among the 48 items showing moderate to perfect agreement, five (10%) failed to demonstrated moderate to perfect agreement in one or both pairwise expert/non-expert values. Two of these five (uneven dirt path on public sidewalk, no light source in bathtub/shower with curtains closed) demonstrated a Krippendorff’s alpha of at least 0.3 in each expert/non-expert comparison and were included in the final item list, while the remaining three (cracked pavement on private path, sloped ground on private path, stair nosing >1.5 inches) did not meet this standard and were excluded, leaving 45 of the original 73 items in the final instrument, for a total of 119 possible hazards per home (reduced from the original instrument that contained 185 total possible hazards). Based on these results, the HEAVI was refined such that 12 of the original 16 objective measures (75%) and 33 of the 57 subjective measures (58%) comprised the final instrument (*p* = 0.22).

**Table 2:** Agreement between graders identifying home hazards: Krippendorff’s alpha (*n* = 20 homes)

Item Class	Criteria for defining graded item as a hazard	Rooms where item graded	# of rooms with item	Three-way comparison	Expert vs Non-Expert Rater 1	Expert vs Non-Expert Rater 2	Non-Expert Rater 1 vs. Non-Expert Rater 2
				*KA value (95% CI)*	*KA value (95% CI)*	*KA value (95% CI)*	*KA value (95% CI)*
Handrails	**Accepted (KA >0.40)**						
	Number of handrails	E, S	2	0.89 (0.75–0.97)	0.91 (0.00–0.91)	0.82 (0.00–0.91)	0.92 (0.00–0.92)
	Handrail perimeter <4 inches or >6.25 inches	E, S	2	0.77 (0.57–0.92)	0.78 (0.00–0.89)	0.86 (0.00–0.86)	0.64 (0.00–0.88)
	Loose handrails	E, S	2	0.75 (0.37–0.94)	0.84 (0.00–0.84)	0.62 (0.00–0.81)	0.78 (0.00–0.78)
	Portion of stairs without handrail	E, S	2	0.64 (0.46–0.79)	0.66 (0.32–0.92)	0.77 (0.00–0.92)	0.52 (0.13–0.90)
	No grab bars in tub/shower	BA	1	0.58 (0.37–0.79)	0.44 (0.00–0.78)	0.90 (0.00–0.90)	**0.36 (−0.07–0.79)**
	No grab bars at toilet	BA	1	0.49 (0.16–0.81)	0.46 (−0.35–0.73)	0.49 (−0.01–0.83)	0.49 (−0.01–0.83)
	**Rejected (KA ≤ 0.40)**						
	No grab bars at sink	BA	1	**0.09 (−0.46–0.55)**	**−0.03 (−1.00–0.49)**	**0.33 (−0.56–0.78)**	**−0.11 (−1.00–0.55)**
	Height of handrail <34 inches or >38 inches	E, S	2	**0.00 (N/A)**	**0.00 (N/A)**	**0.00 (N/A)**	**0.00 (N/A)**
	No contrast between handrails and walls	S	1	**−0.05 (−0.93–0.65)**	**0.00 (−1.00–0.00)**	**−0.04 (−1.00–0.48)**	**−0.09 (−1.00–0.64)**
Floor	**Accepted (KA >0.40)**						
	Cracked external steps	E	1	1.00 (0.00–0.00)	1.00 (0.00–0.00)	1.00 (0.00–0.00)	1.00 (0.00–0.00)
	Height of front step riser <4 inches or >7.75 inches	E	1	0.87 (0.00–0.94)	0.81 (0.00–0.81)	1.00 (0.00–0.00)	0.81 (0.00–0.81)
	Width of stairway <36 inches	E, S	2	0.81 (0.69–0.93)	0.85 (0.00–0.93)	0.85 (0.00–0.93)	0.70 (0.32–0.93)
	Width of hallway <36 inches	H	1	0.79 (0.61–0.93)	0.68 (0.00–0.89)	1.00 (0.00–0.00)	0.68 (0.00–0.89)
	Sloping external steps	E	1	0.78 (0.00–0.89)	1.00 (0.00–0.00)	0.64 (−0.09–0.64)	0.64 (−0.09–0.64)
	Width of doorway <32 inches or >48 inches	E	1	0.78 (0.60–0.93)	0.68 (0.00–0.89)	1.00 (0.00–0.00)	0.68 (0.00–0.89)
	Frayed/torn/folded carpeting	BA, BR, D, H, K, L	6	0.76 (0.56–0.92)	0.71 (0.00–0.85)	0.71 (0.00–0.85)	0.76 (0.00–0.88)
	Height of door threshold >0.5 inches	BA, BR, D, E, H, K, L	7	0.73 (0.59–0.86)	0.82 (0.63–0.96)	0.73 (0.52–0.90)	0.77 (0.58–0.92)
	Loose external steps	E	1	0.73 (0.00–0.87)	0.64 (−0.08–0.64)	1.00 (0.00–0.00)	0.64 (−0.09–0.64)
	Landing more narrow than doorway	E	1	0.72 (0.00–0.86)	1.00 (0.00–0.00)	0.63 (−0.12–0.63)	0.63 (−0.12–0.63)
	Unsecured rugs; rugs with curling edges	BA, BR, D, H, K, L	6	0.68 (0.53–0.82)	0.89 (0.00–0.95)	0.64 (0.39–0.85)	0.58 (0.31–0.84)
	Glossy or shiny finish	BA, BR, D, H, K, L, S	7	0.65 (0.37–0.86)	0.78 (0.61–0.92)	0.48 (0.15–0.77)	0.66 (0.38–0.89)
	Smooth finish (no traction)	E, S	2	0.65 (0.36–0.88)	0.67 (0.00–0.84)	0.40 (−0.20–0.80)	0.76 (0.00–0.76)
	Sloped ground on public sidewalk	E	1	0.62 (0.38–0.86)	0.60 (0.00–0.87)	0.67 (0.00–0.84)	0.58 (0.00–0.86)
	Floor covered with objects/cluttered (>50% clutter)	BA, BR, D, H, K, L	6	0.60 (0.00–0.86)	0.79 (0.00–0.88)	0.56 (0.00–0.84)	0.42 (−0.14–0.87)
	Height of stair riser <4 inches or >7.75 inches	E, S	2	0.60 (0.40–0.78)	0.56 (0.26–0.85)	0.67 (0.35–0.92)	0.61 (0.30–0.84)
	No contrast between door threshold and floor	BA, BR, D, H, K, L	6	0.60 (0.43–0.76)	0.70 (0.46–0.90)	0.56 (0.30–0.78)	0.61 (0.40–0.83)
	Depth of stair treads <10 inches	E, S	2	0.57 (0.38–0.76)	0.45 (0.14–0.77)	0.53 (0.22–0.84)	0.72 (0.00–0.91)
	No contrast between floors and fixtures	E, S	2	0.55 (0.33–0.78)	0.43 (−0.03–0.77)	0.46 (0.04–0.79)	0.73 (0.00–0.87)
	Landing only on one side of doorway	E	1	0.54 (0.00–0.89)	0.63 (−0.111–0.63)	0.63 (−0.12–0.63)	0.63 (−0.12–0.63)
	No non-skid rug/mat outside bathtub/shower	BA	1	0.53 (0.23–0.82)	0.45 (−0.37–0.73)	0.70 (0.00–0.85)	**0.32 (−0.36–0.77)**
	Landing <36 inches	E	1	0.52 (0.21–0.76)	0.65 (0.00–0.83)	0.42 (−0.15–0.81)	0.62 (0.00–0.81)
	No bathmat/non-skid strips in bathtub/shower	BA	1	0.45 (0.18–0.69)	0.53 (0.05–0.88)	0.54 (0.07–0.88)	**0.21 (−0.32–0.74)**
	Uneven dirt path on public sidewalk	E	1	0.41 (0.04–0.78)	0.45 (−0.38–0.72)	**0.30 (−0.39–0.77)**	0.46 (−0.08–0.82)
	**Rejected (KA ≤ 0.40)**						
	Stair nosing >1.5 inches	E	1	0.48 (−0.29–0.74)	**0.00 (−1.00–0.00)**	**0.00 (−1.00–0.00)**	1.00 (0.00–0.00)
	Cracked pavement on private path	E	1	0.46 (0.12–0.80)	**0.18 (−0.43–0.80)**	0.79 (0.00–0.79)	0.44 (−0.41–0.72)
	Sloped ground on private path	E	1	0.46 (0.13–0.80)	1.00 (0.00–0.00)	**0.13 (−0.53–0.78)**	**0.19 (−0.41–0.80)**
	Papers/books/towels/shoes/magazines/boxes/blankets/other objects in pathway	BA, BR, D, H, K, L, S	7	**0.40 (0.09–0.67)**	0.63 (0.31–0.88)	**0.26 (−0.03–0.54)**	**0.39 (0.09–0.64)**
	Broken tiles	BA, BR, D, H, K, L	6	**0.38 (−0.16–0.87)**	**−0.01 (−1.00–0.49)**	0.48 (−0.31–0.74)	**−0.02 (−1.00–0.49)**
	Exterior door swings over step with no landing	E	1	**0.38 (0.01–0.69)**	0.59 (−0.02–0.80)	**0.11 (−0.59–0.65)**	0.58 (−0.04–0.79)
	No contrast between stair treads and risers	E, S	2	**0.37 (−0.16–0.79)**	**0.00 (−1.00–0.00)**	**−0.02 (−1.00–0.49)**	0.47 (−0.33–0.74)
	Uneven dirt path on private path	E	1	**0.34 (−0.21–0.78)**	**−0.04 (−1.00–0.48)**	**−0.12 (−1.00–0.63)**	**0.77 (0.00–0.77)**
	Cracked pavement on public sidewalk	E	1	**0.32 (−0.08–0.66)**	**0.21 (−0.60–0.80)**	0.45 (−0.10–0.82)	0.44 (−0.41–0.72)
	Loose bricks or paver stones on public sidewalk	E	1	**0.30 (−0.40–0.83)**	**0.00 (−1.00–0.00)**	0.64 (−0.08–0.64)	**−0.04 (−1.00–0.48)**
	Loose electrical/phone cords on floor	BA, BR, D, H, K, L	6	**0.28 (−0.22–0.72)**	**−0.02 (−0.97–0.75)**	0.50 (0.07–0.85)	**0.09 (−0.49–0.59)**
	No contrast between floors and furniture/obstacles	BR, D, K, L	4	**0.19 (−0.13–0.45)**	**0.18 (−0.13–0.48)**	**0.36 (0.04–0.68)**	**−0.14 (−0.61–0.33)**
	No contrast between stair treads	E, S	2	**0.16 (−0.47–0.69)**	**0.00 (−1.00–0.00)**	**−0.06 (−1.00–0.74)**	**0.35 (−0.51–0.78)**
	Walkway narrowed by obstacles/furniture	BA, BR, D, K, L	5	**0.15 (−0.64–0.82)**	**0.38 (−0.44–0.79)**	**−0.02 (−1.00–0.66)**	**0.00 (−1.00–0.00)**
	Blankets/sheets on floor	BR	1	**0.09 (−0.46–0.54)**	**0.33 (−0.35–0.78)**	**−0.09 (−1.00–0.73)**	**−0.06 (−1.00–0.65)**
	Slippery floor (no traction with soled shoes)	BA, BR, D, H, K, L	6	**0.01 (−0.37–0.34)**	**0.08 (−0.22–0.37)**	**−0.18 (−0.60–0.20)**	**0.11 (−0.46–0.64)**
	Carpet runners with curling edges	BA, BR, D, H, K, L	6	**0.00 (−0.67–0.50)**	**0.00 (N/A)**	**0.00 (−1.00–0.00)**	**−0.29 (−1.00–0.57)**
	Interior door swings over step with no landing	E	1	**0.00 (−1.00–0.50)**	**0.00 (−1.00–0.00)**	**0.00 (−1.00–0.00)**	**0.00 (N/A)**
	Loose/torn coverings on treads	S	1	**0.00 (N/A)**	**0.00 (N/A)**	**0.00 (N/A)**	**0.00 (N/A)**
	Depth of front step tread <10 inches	E	1	**0.00 (N/A)**	**0.00 (N/A)**	**0.00 (N/A)**	**0.00 (N/A)**
	Loose bricks or paver stones on private path	E	1	**−0.02 (−1.00–0.74)**	**0.00 (N/A)**	**−0.06 (−1.00–0.47)**	**−0.04 (−1.00–0.48)**
Lighting	**Accepted (KA >0.40)**						
	Broken light fixture/missing bulbs	E, S	2	1.00 (0.00–0.00)	1.00 (0.00–0.00)	1.00 (0.00–0.00)	1.00 (0.00–0.00)
	Missing light fixture	E, S	2	0.78 (0.61–0.92)	0.85 (0.00–0.92)	0.76 (0.00–0.88)	0.71 (0.00–0.90)
	Exposed light bulbs	BA, BR, D, H, K, L, S	7	0.76 (0.62–0.88)	0.77 (0.64–0.88)	0.74 (0.60–0.86)	0.75 (0.62–0.88)
	Shadows on stairway	E, S	2	0.76 (0.61–0.88)	0.72 (0.37–0.93)	0.85 (0.00–0.92)	0.70 (0.32–0.92)
	Ambient light <300 lux	BA, BR, D, E, H, K, L, S	8	0.67 (0.46–0.87)	0.58 (0.36–0.80)	0.63 (0.41–0.82)	0.83 (0.68–0.97)
	Less than two light switches	S	1	0.66 (0.24–0.92)	1.00 (0.00–0.00)	0.43 (−0.42–0.72)	0.43 (−0.42–0.72)
	Switch required to turn on exterior light	E	1	0.66 (0.45–0.87)	0.87 (0.00–0.87)	0.48 (−0.03–0.87)	0.62 (0.00–0.87)
	Windows without sheer coverings	BA, BR, D, H, K, L, S	7	0.53 (0.36–0.68)	0.41 (0.24–0.58)	0.65 (0.48–0.79)	0.49 (0.32–0.64)
	Light switches only accessible from fully inside room	BA, BR, D, H, K, L	6	0.52 (0.32–0.70)	0.52 (0.32–0.71)	0.53 (0.31–0.71)	0.51 (0.30–0.71)
	No light source in bathtub/shower with curtains closed	BA	1	0.41 (0.18–0.64)	**0 .39 (−0.02–0.80)**	0.51 (0.12–0.81)	**0.32 (−0.08–0.71)**
	**Rejected (KA ≤ 0.40)**						
	No nightlight/flashlight near bed or for nighttime bathroom use	BR	1	**0.29 (0.09–0.50)**	**0.38 (0.03–0.72)**	**0.22 (−0.12–0.55)**	**0.21 (−0.11–0.52)**
	No light on path from bedroom to bathroom	BR	1	**0.12 (−0.19–0.43)**	**0.33 (−0.35–0.78)**	**−0.28 (−0.84–0.29)**	**0.14 (−0.29–0.57)**
Furniture	**Accepted (KA >0.40)**						
	Seats without arm rests	BR, D, K, L	4	0.63 (0.48–0.78)	0.75 (0.57–0.89)	0.55 (0.33–0.78)	0.60 (0.39–0.82)
	Glossy/shiny finish of countertop	K	1	0.50 (0.24–0.71)	0.48 (0.06–0.79)	0.48 (0.06–0.79)	0.54 (0.07–0.88)
	Unstable furniture	BR, D, K, L	4	0.41 (0.15–0.64)	0.51 (0.22–0.76)	0.48 (0.19–0.72)	**0.16 (−0.26–0.52)**
Other	**Accepted (KA >0.40)**						
	No handle on step stool	BR, K	2	0.67 (0.47–0.83)	0.91 (0.00–0.91)	0.51 (0.00–0.84)	0.40 (−0.20–0.85)
	Inability to reach microwave with both feet on floor	K	1	0.57 (0.03–0.89)	0.65 (−0.06–0.65)	0.46 (−0.36–0.73)	0.65 (−0.06–0.65)
	**Rejected (KA ≤ 0.40)**						
	Step stool required to get onto bed	BR	1	**0.00 (N/A)**	**0.00 (N/A)**	**0.00 (N/A)**	**0.00 (N/A)**
	Inability to reach closet items or daily kitchen items with both feet on floor	BR, K	2	**−0.06 (−0.69–0.47)**	**−0.04 (−1.00–0.74)**	**−0.06 (−0.90–0.79)**	**−0.07 (−0.96–0.64)**
**Total number of possible hazards per home**		**185**					
Accepted		119					
Rejected		66					

BA = bathroom, BR = bedroom, D = dining room, E = entry way, H = hallway, K = kitchen, L = living room, S = stairs, KA = Krippendorff’s alpha, CI = confidence interval

KA categories: 0.81–1.00 = almost perfect; 0.61–0.80 = substantial; 0.41–0.60 = moderate; 0.21–0.40 = fair; 0.00–0.20 = slight; <0.00 = poor ^39^

Bold values indicate inadequate agreement

### Assessment of interrater agreement

Using this final instrument, the average number of hazards identified per home did not differ by grader. Out of a total of 119 possible hazards per home, an average of 35.7 (SD = 9.1) were identified by the expert grader, and 33.2 (SD = 9.5) and 32.3 (SD = 8.9) for the two non-expert graders (*p* >0.20 for each pair-wise comparison of expert and non-expert graders) (Table 3). There were no significant differences in the number of hazards identified in each of the eight rooms by the expert and each non-expert grader (*p* >0.20 for all comparisons).

**Table 3:** Mean and standard deviation of the number of hazards by room and grader (*n* = 20 homes)

	Expert		Non-expert 1		Non-expert 2	
Room (# possible hazards)	Mean # identified hazards (SD)	% of items Graded as hazard	Mean (SD)	% of items Graded as hazard	Mean (SD)	% of items Graded as hazard
Living Room (12 items)	3.3 (1.8)	27.5%	3.1 (1.6)	25.8%	3.1 (1.8)	25.8%
Dining Room (12 items)	3.5 (1.9)	29.2%	3.4 (2.7)	28.3%	3.3 (1.5)	27.5%
Kitchen (15 items)	4.9 (1.7)	32.7%	4.2 (1.4)	28.0%	4.3 (1.7)	28.7%
Bedroom (13 items)	4.2 (1.9)	32.3%	3.5 (1.8)	26.9%	3.8 (1.6)	29.2%
Bathroom (16 items)	7.4 (2.7)	46.3%	7.1 (2.3)	44.4%	6.6 (2.6)	41.3%
Hallway (11 items)	3.1 (1.9)	28.2%	2.8 (1.8)	25.5%	3.0 (1.5)	27.3%
Stairs (16 items)	4.5 (3.3)	28.1%	4.2 (3.0)	26.3%	3.3 (2.8)	20.6%
Entrance (24 items)	5.1 (3.4)	21.3%	5.0 (3.2)	20.8%	5.0 (3.4)	20.8%
Entire Home (119 items)	35.7 (9.1)	30.0%	33.2 (9.5)	27.9%	32.3 (8.9)	27.1%

SD = standard deviation

All comparisons between the expert and each non-expert were not significant (*p* <0.05)

Agreement on the total number of hazards per room and per home was evaluated using the final instrument. Agreement on the total number of hazards per home was 0.83 across all three graders; pairwise agreement between the expert and each non-expert grader were 0.84 and 0.81, and agreement between the two non-expert graders was 0.85, indicating almost perfect agreement for each comparison (ICC >0.81) (Table 4). Likewise, agreement on the number of hazards per room was substantial to almost perfect (ICC >0.61) for all three-way comparisons (Table 4). In pairwise comparisons of the number of hazards per room, moderate to perfect agreement (>0.40) was observed for all rooms, except for pairwise comparisons between each of the non-expert graders to the expert grader for dining room, where agreement was fair (<0.41).

**Table 4:** Consistency of number of hazards by room and home after removing hazards with poor agreement (*n* = 20 homes)

Hazard	Absolute Agreement ICC							
	All Raters		Expert vs. non-expert 1		Expert vs. non-expert 2		Non-expert 1 vs. non-expert 2	
	ICC (95% CI)	*P* value	ICC (95% CI)	*P* value	ICC (95% CI)	*P* value	ICC (95% CI)	*P* value
Living Room	0.68 (0.46,0.85)	**<0.001**	0.68 (0.35,0.86)	**<0.001**	0.62 (0.26,0.83)	**<0.001**	0.75 (0.47,0.90)	**<0.001**
Dining Room	0.43 (0.15,0.70)	**<0.001**	0.31 (−0.16,0.66)	0.094	0.37 (−0.08,0.70)	0.053	0.67 (0.33,0.86)	**0.001**
Kitchen	0.64 (040,0.82)	**<0.001**	0.68 (0.31,0.87)	**<0.001**	0.65 (0.31,0.84)	**<0.001**	0.58 (0.18,0.81)	**0.004**
Bedroom	0.70 (0.50,0.86)	**<0.001**	0.66 (0.31,0.85)	**0.001**	0.76 (0.50,0.90)	**<0.001**	0.71 (0.41,0.87)	**<0.001**
Bathroom	0.83 (0.67,0.92)	**<0.001**	0.82 (0.61,0.93)	**<0.001**	0.83 (0.57,0.93)	**<0.001**	0.83 (0.62,0.93)	**<0.001**
Hallway	0.78 (0618,0.90)	**<0.001**	0.76 (0.49,0.90)	**<0.001**	0.81 (0.59,0.92)	**<0.001**	0.79 (0.55,0.91)	**<0.001**
Stairs	0.79 (0.60,0.90)	**<0.001**	0.92 (0.80,0.96)	**<0.001**	0.70 (0.34,0.87)	**<0.001**	0.75 (0.44,0.89)	**<0.001**
Entrance	0.91 (0.82,0.96)	**<0.001**	0.93 (0.83,0.97)	**<0.001**	0.90 (0.77,0.96)	**<0.001**	0.89 (0.75,0.96)	**<0.001**
Entire home	0.83 (0.67,0.93)	**<0.001**	0.84 (0.60,0.93)	**<0.001**	0.81 (0.42,0.93)	**<0.001**	0.85 (0.66,0.94)	**<0.001**

ICC = intraclass coefficient; CI = confidence interval

ICC agreement categories: 0.81–1.00 = almost perfect; 0.61–0.80 = substantial; 0.41–0.60 = moderate; 0.21–0.40 = fair; 0.00–0.20 = slight; <0.00 = poor ^39^

Bold values indicate significant results

**Fig. 1:**
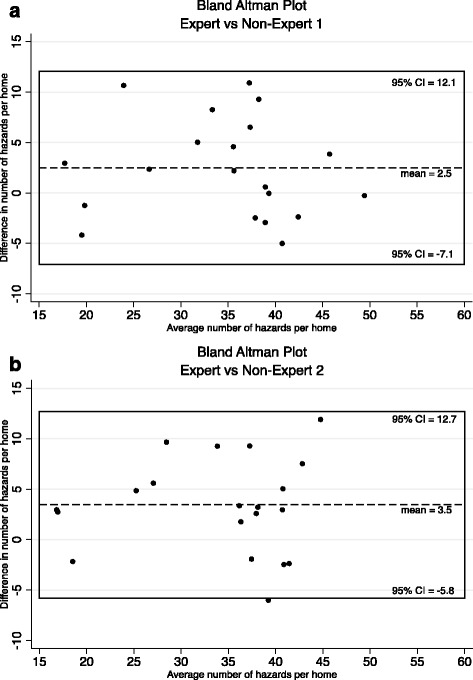
**a** Bland Altman plot comparing the expert grader and non-expert grader 1. **b** Bland Altman plot comparing the expert grader and non-expert grader 2

Differences in the number of hazards identified by the expert and each non-expert grader are shown in Bland-Altman plots (Fig. 1a and b). On average, there was no significant difference between the number of hazards identified by the expert grader as compared to the non-expert graders (non-expert 1 vs. expert: +2.5, 95% CI =−7.1 – 12.1, *p* = 0.40; non-expert 2 vs. expert: +3.5, 95% CI =−5.8 – 12.7, *p* = 0.23). Additionally, the difference in the number of hazards identified by the non-expert and expert graders did not vary across the observed range for average number of hazards identified by each expert/non-expert pair (*p* = 0.73 for Fig. 1a and *p* = 0.91 for Fig. 1b).

### Home assessment results

We also examined our data to identify which home locations and item classes were most hazardous. Of the rooms graded, the bathroom was found to be the most hazardous as judged by the total number of hazardous items and the percentage of items graded as hazardous (Table 3). There was a total of 16 items graded in the bathroom for each home, and the mean number classified as hazardous ranged from 6.6 to 7.4 for all three graders. Of the five types of hazards (handrails, floors, lighting, furniture, and other, as shown in Table 2), all three graders most often identified items related to handrails and lighting as hazardous. There were 10 handrail items graded per home, and the mean number graded as hazardous was ≥3.1 (>30%) for all three graders. Of the 37 lighting items graded per home, the mean number identified as hazardous was ≥11.5 (>30%) by all three graders. Additionally, all three graders reported that ≥60% of rooms had less than 300 lux of ambient light (the classification of ambient lighting hazard). All three graders also found that most rooms had an average ambient lighting level below 300 lux across the whole home, with the bathroom serving as an exception (where average lighting was measured as >321 lux by all three graders).

## Discussion

Here, we develop and refine the HEAVI tool, a novel instrument designed to: (1) assess fall-related hazards specific to older adults with visual impairments; (2) be administered by a non-expert grader evaluating the home environment; (3) incorporate several elements regarding lighting, contrast, and changes in elevation which may be particularly hazardous for persons with poor vision; and (4) objectively measure home features when possible. While 73 items were initially evaluated as part of this screening tool, only 45 of these demonstrated acceptable agreement between an expert and two non-expert graders. Many of these items were graded in multiple rooms, and therefore the original instrument, which included a total of 185 potential hazards, was reduced to 119 in this final instrument. When these final 45 items were assessed, good agreement was noted between the expert and non-expert grader with regard to overall number of home hazards and the number of hazards in each of the eight rooms graded. A larger proportion of objective items was retained in the final instrument as compared to subjective items, but this difference was not significant (*p* = 0.22) suggesting a trend towards objective measures being more reliably graded.

Several other home assessments have been previously validated,[25–29] though the current instrument was designed to fill a specific niche. First, it was primarily geared towards the visually impaired, with an emphasis on lighting, contrast, and other items which could generate particular problems for those with visual impairment. It is quite possible that these same hazards would pose risks to persons without visual impairment as well – a point which requires further study. It is unclear which specific types of visual impairment would be best served by the HEAVI, though multiple visual measures including visual field loss, contrast sensitivity, stereo acuity, and visual acuity have all been associated with higher rates of falls,[14–24] suggesting that most eye diseases will produce higher rates of falls which could potentially be modified via a safer home environment. Second, the instrument was designed to be geared specifically to fall-related hazards and did not evaluate other home hazards that may also be relevant to the visually impaired (i.e. dangers related to food preparation). Finally, the assessment tool was designed to be administered by a person without specific skills in home assessments, as most low vision services are delivered by persons without expertise in modifying homes to reduce fall risk.

Our results indicate that non-expert graders can reliably administer this home assessment tool, as we observed good interrater agreement. We observed substantial to almost perfect agreement (>0.60) between all three graders for the number of hazards found in all rooms, with the exception of the dining room where agreement was moderate (0.41 – 0.60) (Table 4). Agreement on the number of hazards in dining rooms was almost perfect between the two non-expert graders, which suggests that while the non-expert graders had a low level of agreement with the expert grader, their ratings were consistent with each other. The items graded in the dining room that had fair to poor agreement between the expert and each non-expert grader (ICCs ranging from −0.13 to 0.36), but moderate agreement between the two non-expert graders (ICCs ranging from 0.47 to 0.54) were assessments of windows without sheer coverings and exposed light bulbs. High agreement (>0.80) was observed for the number of bathroom, entrance, and total home hazards found by the three graders (Table 4). This high level of agreement between graders may reflect the inclusion of objectively graded items (such as stair riser height and number of handrails) not present in other existing home assessments. Additionally, the non-expert graders received training from the same expert grader to whom they were being compared. Only two homes (not included in this study) were used for training, though it is possible that some degree of training from a home-assessment professional may be needed to achieve the high level of interrater agreement in future use of the tool.

We did not find a difference between the total number of hazards rated between the expert and the two non-expert graders (Fig. 1a and b). Additionally, differences in the number of graded hazards were not influenced by the presence of high or low number of hazards within a given home. Over the eight rooms graded, the observed differences amount to less than one hazard per room. While this difference was not statistically significant, it is difficult to determine the practical implications of this difference from these analyses, as each potential hazard may pose a different level of fall risk. It is possible that additional training, for example increasing training to three or more homes, may reduce this difference.

In addition to creating a final instrument, we examined the types and location of hazards found in this study population. The room with the highest percentage of items graded as hazardous was the bathroom (Table 3), while handrail and lighting items were most often hazardous in the entire home. These results suggest that these areas/classes of items may be particularly important when addressing falls. Poor ambient room lighting (<300 lux) was among the most commonly reported hazards, and only lighting in the bathroom was, on average, greater than 300 lux. This suggests that inadequate lighting is a pervasive in-home factor contributing to falls, especially among those with vision loss. As lighting is an easily modifiable environmental condition, this study provides preliminary evidence that home lighting is an important intervention target.

There are limitations to this study that need to be considered. First, assessments were only made at one time point, and therefore our reliability analyses do not assess parameters of individual grader repeatability. Second, a convenience sample of participants that included those with and without visual impairment was used to evaluate this instrument. While we do not expect the performance of the in-home assessment tool to differ by degree and type of vision loss, this will need to be explored further in a larger study population. Third, this instrument does not include an assessment of the individual’s physical or mental abilities, and therefore does not assess the individual’s interaction with their environment. It is possible that some identified hazards may not be hazardous for a particular individual, or vice versa. Fourth, we compared two non-expert graders to one expert for these analyses. To determine the clinical utility of this instrument, further comparisons with a broader group of graders (both expert and non-expert) will be required. Fifth, we only focused on one owner/occupant of the home given the logistical challenges of getting consent from all occupants, which may bias the demographic data that was collected. However, the intent of this work was not to characterize the owners of the home, but to see if the graders could grade homes similarly. Sixth, the items added to the HEAP were added largely based on the experience of the OT (VG), though additions were supported by a systematic review of the literature. Additionally, the expert grader who helped to develop the HEAVI also trained the non-expert graders. It is possible that this could introduce bias, but it is unknown if our expert grader is superior to other OTs in getting non-expert graders to produce the same grading. Lastly, this study does not determine how the number of hazards relates to fall risk; however, this work is currently underway, and prior studies have found a relationship between home hazards and risk of home injury [12, 41].

While the developed home assessment tool was based on a prior instrument, the HEAP, this assessment tool is unique. The final assessment includes 10 items assessing lighting and two items assessing contrast between objects. Both are particularly important for those with vision loss,[32, 33] and lack of appropriate lighting and contrast places those with visual impairment at greater risk of falling. Additionally, this tool includes a greater emphasis on objective measurements, which are typically not evaluated in existing home assessments. Inclusion of objective measures was shown to facilitate agreement and repeatability between graders, which in turn may reduce the need for specialized expertise to accurately administer this in-home assessment tool. Lastly, this instrument is intended to grade the home, and not the person within the home. To that extent, it may be relevant when setting up residences for older adults (where the person living there may not be known) and for common spaces where multiple people will be living.

## Conclusion

The development of this tool has important clinical implications. While multiple in-home fall hazard assessments have been developed, none have specifically included items that assess fall risk among those with vision loss. Both the high rate of falls and the increasing number of visually impaired older adults highlight the need for home assessment tools to better capture fall hazards that are specifically problematic for individuals with vision impairment. Additionally, risk factors important for those with vision loss may also impact older adults with normal or near-normal vision, such as poor visual perception due to cognitive decline.[42, 43] Further research will explore if this assessment is associated with greater risk of in-home falls in a visually impaired population and help determine which specific home features increase fall risk in older adults.

## Abbreviations

CI: Confidence interval
HEAP: Home environmental assessment protocol
HEAVI: Home environment assessment for the visually impaired
ICC: Intraclass correlation coefficients
OT: Occupational therapist
SD: Standard deviation
